# Inhibition mechanisms of hemoglobin, immunoglobulin G, and whole blood in digital and real-time PCR

**DOI:** 10.1007/s00216-018-0931-z

**Published:** 2018-03-05

**Authors:** Maja Sidstedt, Johannes Hedman, Erica L. Romsos, Leticia Waitara, Lars Wadsö, Carolyn R. Steffen, Peter M. Vallone, Peter Rådström

**Affiliations:** 1 Applied Microbiology, Department of Chemistry, Lund University, P.O. Box 124, 221 00 Lund, Sweden; 2 Swedish National Forensic Centre, 581 94 Linköping, Sweden; 3 Materials Measurement Laboratory, National Institute of Standards and Technology, Gaithersburg, MD 20899-8314 USA; 4 Present Address: Government Chemist Laboratory Authority, P.O. Box 164, Dar es Salaam, Tanzania; 5 Division of Building Materials, Lund University, 221 00 Lund, Sweden

**Keywords:** Blood, DNA polymerase, Digital PCR, PCR inhibition, PCR inhibitors, Real-time PCR

## Abstract

Blood samples are widely used for PCR-based DNA analysis in fields such as diagnosis of infectious diseases, cancer diagnostics, and forensic genetics. In this study, the mechanisms behind blood-induced PCR inhibition were evaluated by use of whole blood as well as known PCR-inhibitory molecules in both digital PCR and real-time PCR. Also, electrophoretic mobility shift assay was applied to investigate interactions between inhibitory proteins and DNA, and isothermal titration calorimetry was used to directly measure effects on DNA polymerase activity. Whole blood caused a decrease in the number of positive digital PCR reactions, lowered amplification efficiency, and caused severe quenching of the fluorescence of the passive reference dye 6-carboxy-X-rhodamine as well as the double-stranded DNA binding dye EvaGreen. Immunoglobulin G was found to bind to single-stranded genomic DNA, leading to increased quantification cycle values. Hemoglobin affected the DNA polymerase activity and thus lowered the amplification efficiency. Hemoglobin and hematin were shown to be the molecules in blood responsible for the fluorescence quenching. In conclusion, hemoglobin and immunoglobulin G are the two major PCR inhibitors in blood, where the first affects amplification through a direct effect on the DNA polymerase activity and quenches the fluorescence of free dye molecules, and the latter binds to single-stranded genomic DNA, hindering DNA polymerization in the first few PCR cycles.

**Electronic supplementary material:**

The online version of this article (10.1007/s00216-018-0931-z) contains supplementary material, which is available to authorized users.

**Graphical abstract:**
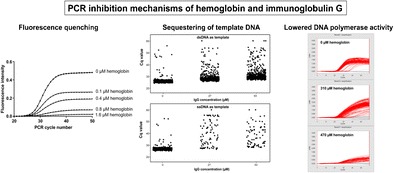
PCR inhibition mechanisms of hemoglobin and immunoglobulin G (IgG). Cq quantification cycle, dsDNA double-stranded DNA, ssDNA single-stranded DNA

## Introduction

Blood samples are widely used for polymerase chain reaction (PCR) analysis in fields such as diagnosis of infectious and genetic diseases in clinical medicine and forensic genetics. Direct PCR analysis with blood, without prior DNA extraction and purification, has been attempted to save time and reduce costs in routine analysis [1–6]. This approach is promising but is still limited by PCR inhibition induced by blood compounds [7, 8]. In 1988, it was noted that *Taq* DNA polymerase was affected by a substance co-purified with DNA in extracts prepared from human blood [9]. Early on, a heme compound was implicated as an inhibitor in blood [10]. To bypass inhibition by blood, researchers have screened for robust DNA polymerases or engineered enzymes to improve compatibility with the inhibitors encountered in blood, and have identified facilitators that may allow amplification in the presence of blood components [11–14].

PCR inhibitors may affect amplification by lowering or even blocking the DNA polymerase activity or by interacting with the nucleic acids (i.e., DNA template or primers) [15]. We recently identified another mode of inhibition: quenching of fluorescence, leading to failed detection of amplicons [16]. The main amplification inhibitors in human whole blood are hemoglobin and immunoglobulin G (IgG) [8, 17]. Hemoglobin disturbs DNA polymerase activity, as shown by great differences in hemoglobin tolerance between different DNA polymerases [8]. Each hemoglobin molecule contains four heme groups, which contain iron, and hence the ability to release iron has been suggested to be the reason why hemoglobin and blood inhibit PCR [8]. IgG has been implicated as the cause of amplification inhibition by blood plasma [17]. This is likely a general immunoglobulin effect, and not connected with specific clones. IgG was suggested to act on single-stranded DNA (ssDNA), as the effect was partly counteracted by addition of nontarget lambda DNA and as inhibition was severer when IgG and target DNA were heated together before PCR [17].

Previous work on elucidating PCR inhibition mechanisms of blood components was mainly performed by use of conventional PCR with gel electrophoresis [8, 10, 17]. Other PCR-based technologies, such as real-time PCR (qPCR) and digital PCR (dPCR), may be affected in different ways, for example, because of different detection principles. Also, more information related to mechanisms may be acquired through the quantitative real-time measurements of qPCR and dPCR. The continuous development of inhibitor-tolerant DNA polymerases has improved the ability to analyze impure samples, possibly leading to new bottlenecks in the analysis, adding to the need to study PCR inhibition mechanisms in a modern context.

The objective of this study was to investigate the mechanisms behind PCR inhibition by blood and gain a greater understanding of how blood disturbs the reaction. To that end, qPCR and dPCR were combined with electrophoretic mobility shift assay (EMSA) and isothermal titration calorimetry (ITC) experiments. Apart from amplification inhibition, fluorescence quenching effects of blood and blood components were studied in qPCR and dPCR. In PCR experiments, it is difficult to separate inhibitor effects related to DNA polymerase activity from those connected with DNA interactions as the analysis success is determined by a combination of several subreactions. Therefore, possible binding between DNA and proteins was studied by EMSA, and ITC was applied to directly measure the impact of blood compounds on DNA polymerase activity. Notably, by examination of whole blood as well as some of the major molecular inhibitors (IgG, hemoglobin, hematin, and iron trichloride) it was possible to obtain enhanced understanding of the complexity of inhibition in the analysis of blood samples. The increased knowledge regarding PCR inhibition mechanisms may be applied in the development of more robust PCR systems for direct PCR analysis of blood samples.

## Materials and methods

Throughout the study, two assays were applied: one was a human-specific assay targeting the retinoblastoma 1 gene (*RB1*) with an amplicon size of 156 bp [18] and the other was an assay targeting the *Salmonella enterica* serovar Typhimurium (*Salmonella* Typhimurium) *invA* gene with an amplicon size of 88 bp [19]. The DNA polymerase used, Ex*Taq* HS (TaKaRa Bio, Shiga, Japan), has been shown to be robust to inhibitors in saliva traces and humic acid [16, 20]. The amount of DNA was kept constant in the experiments to evaluate only the effects of the inhibitors.

### Materials

#### DNA

The human genomic DNA for the *RB1* assay in dPCR was a material derived from an in-house standard that was described in previous reports [21, 22]. The genomic DNA for the *RB1* assay in qPCR was extracted from blood from one male by Chelex-based extraction [23]. The DNA concentration was measured with the Qubit dsDNA BR assay (Thermo Fisher Scientific) with use of a Qubit 3.0 fluorometer (Thermo Fisher Scientific). The genomic DNA for the *invA* assay was prepared in the following way: *Salmonella* Typhimurium strain CCUG 31969 was regenerated from a -80 °C glycerol stock by our streaking it on a brain–heart infusion agar plate (Difco Laboratories, BD Diagnostic Systems, NJ, USA) and incubated overnight at 37 °C. Thereafter, a single colony was transferred to liquid Luria–Bertani medium and incubated at 37 °C until an optical density at 600 nm of approximately 1 was reached. Thereafter a GeneJET genomic DNA purification kit (Thermo Fisher Scientific, Waltham, MA, USA) was used for isolation of the genomic DNA. The concentration was measured with the Qubit dsDNA BR assay as described above. All extracts were stored at -20 °C. For preparation of ssDNA template, *Salmonella* Typhimurium genomic DNA was heated for 5 min at 95 °C and human genomic DNA was heated for 15 min at 95 °C with use of an Applied Biosystems GeneAmp PCR system 9700 thermal cycler (Thermo Fisher Scientific) and then flash-cooled and used immediately. Amplicons for use in the EMSA analysis were prepared by our purifying PCR products from the *invA* assay performed in 50-μL reaction mixtures with an Applied Biosystems GeneAmp PCR system 9700 thermal cycler. The PCR products were purified with use of a QIAquick gel extraction kit (Qiagen, Hilden, Germany), and the concentration was measured with the Qubit dsDNA BR assay as described above.

#### Inhibitors

IgG (product number I4506) was purchased from Sigma-Aldrich (Taufkirchen, Germany) and dissolved in water to a stock concentration of 80 μg/μL. Human hemoglobin was purchased from Sigma-Aldrich (product number H7379) and dissolved in water to a stock concentration of 100 μg/μL. Porcine hematin was purchased from Sigma-Aldrich (product number H3281) and dissolved in 0.1 M NaOH to form a 6 mM stock solution. FeCl_3_ was purchased from Spectrum Chemical Manufacturing (New Brunswick, NJ, USA) and dissolved in water to 0.1 M. Subsequent dilutions of the inhibitors were made with water. For comparison purposes, the final concentrations of inhibitors in the reactions were calculated on the basis of the molecular mass or molar mass of IgG (150 kDa), hemoglobin (64.5 kDa), hematin (633.49 g/mol), and FeCl_3_ (162.2 g/mol).

In dPCR, the following concentrations of the inhibitors were tested in triplicate: (1) 2.5%, 5.0%, 7.5%, 10%, and 15% (v/v) blood; (2) 27, 40, 53, 67, 110, 130, 160, and 190 μM IgG; (3) 39, 78, 160, 310, 470, and 620 μM hemoglobin; (4) 1000, 2000, 3000, 4000, and 6000 μM hematin; (5) 1000, 2000, 3000, 4000, and 6000 μM FeCl_3_.

In qPCR, the following concentrations of the inhibitors were tested in triplicate: (1) 0.00005%, 0.0005%, 0.005%, 0.05%, 1%, 5%, 10%, and 20% (v/v) blood; (2) 1.7, 3.3, 6.7, 13, 27, 33, and 53 μM IgG: (3) 0.1, 0.4, 0.8, 1.6, 16, 62, 120, 160, 310, 470, and 620 μM hemoglobin; (4) 1, 5, 10, 15, 25, 50, 60, and 80 μM hematin; (5) 1, 2, 5, 10, 20, 30, and 40 μM FeCl_3_.

#### Blood

Whole blood used in this work came from an anonymous donor and was collected in a Vacuette K_3_EDTA tube (product number 454021, Greiner Bio-One International, Kremsmünster, Austria). The tubes were spray-dried with 1.2–2.0 mg EDTA per milliliter of blood, corresponding to approximately 4 mM EDTA in the blood. For 15% whole blood in the PCR, there will be approximately 0.6 mM EDTA in the reactions. We assessed the inhibitory effect of EDTA and found that 2 mM did not disturb the reaction, whereas 4 mM EDTA resulted in complete amplification inhibition in dPCR with the *invA* assay (data not shown), meaning that the EDTA in the blood is not expected to impact amplification.

### Digital PCR

All dPCR experiments were performed with a BioMark 48.770 Digital Array real time/end point limiting dilution assay system (Fluidigm, San Francisco, CA, USA). The Fluidigm Digital PCR Analysis tool provided by the manufacturer was used for all primary data reduction with assay-specific global intensity thresholds and a quality score threshold of 0.1. Cycles 1–60 were analyzed with the user global analysis method for determination of the positive chambers. Detailed results were exported as .csv files for further data handling.

The primers and hydrolysis probes (purchased from Thermo Fisher Scientific) used in this study target either the *invA* gene of *Salmonella* Typhimurium DNA or the *RB1* gene of human DNA. The probes used in this study were labeled with 6-carboxyfluorescein. The passive reference dye 6-carboxy-X-rhodamine (ROX) (Thermo Fisher Scientific) was used for normalization.

For the *invA* assay, the amplification conditions were 95 °C for 2 min, followed by 60 cycles of 10 s at 95 °C, 20 s at 60 °C, and 30 s at 72 °C. The ramp speed between temperature set points was 2 °C/s. Unless otherwise stated, the following reagents were included in all master mixes for the *invA* assay: 1× Ex*Taq* buffer (TaKaRa Bio), 0.2 mM deoxynucleoside triphosphate (dNTP; Roche Diagnostics, Basel, Switzerland), 2.0 mM MgCl_2_ in total (Roche Diagnostics), each primer at 0.3 μM (InvA forward primer, InvA reverse primer [19]), 0.2 μM hydrolysis probe (InvA-minor groove binder [19]), 1.0 μL of 20× GE loading reagent (Fluidigm), 1 U Ex*Taq* HS DNA polymerase (TaKaRa Bio), 1× ROX, and 2 μL DNA (approximately 30 pg/μL).

For the *RB1* assay, the amplification conditions were 95 °C for 2 min, followed by 60 cycles of 15 s at 95 °C and 1 min at 60 °C. The ramp speed between temperature set points was 2 °C/s. Unless otherwise stated, the following reagents were included in all master mixes for the *RB1* assay: 1× Ex*Taq* buffer, 0.2 mM dNTP, 4.0 mM MgCl_2_ in total, each primer at 0.3 μM (RB1_80F and RB1_235R [18]), 0.2 μM hydrolysis probe (RB1_212_MGB [18]), 1.0 μL of 20× GE loading reagent, 1 U Ex*Taq* HS DNA polymerase, 1× ROX, and 2 μL DNA (approximately 25 ng/μL).

For the inhibitor experiments, the inhibitory compound was added to a master mix containing all the reagents described above, including DNA. If needed, amplification-grade water (Promega, Madison, WI, USA) was added to obtain a total volume of 20 μL. This volume was then used for triplicate dPCR analyses by adding 4-µL aliquots of the prepared master mixes to the appropriate sample inlet for each panel of a 48.770 array. The arrays were filled with use of a BioMark IFC controller MX and placed into the BioMark system for amplification and detection.

### Data analysis of dPCR results

Data were analyzed with the Fluidigm Digital PCR Analysis software program (version 3.1.3, build 20120816.1505) to determine the number of positive chambers. The calculation of DNA concentrations, determination of quantification cycle (Cq) values and amplification efficiency, and analysis of ROX fluorescence intensity were performed as described in a previous study [22]. Briefly, we used the equation suggested by Dorazio and Hunter [24] to calculate the concentration of DNA (copies per microliter):

$$ \left[\mathrm{DNA}\right]=\frac{-\ln \left[1-\left(y/770\right)\right]}{0.75\times {10}^{-3}}, $$

where *y* is the number of positive chambers, 0.75 × 10^-3^ is the nominal chamber volume (in microliters) reported by the manufacturer, and 770 is the total number of chambers in each panel. Determination of original sample concentration for the human-specific *RB1* assay (ng/μL) was performed by our multiplying the number of copies per microliter by 10 (dilution factor) and dividing by 311 (approximate number of copies per nanogram, assuming 6.436 pg DNA per diploid human male cell [25]).

Determination of original sample concentration (pg/μL) for *Salmonella* Typhimurium was performed by multiplying the number of copies per microliter by 10 (dilution factor) and dividing by 200 (approximate number of copies per picogram, assuming a weight of 5 fg for one copy of *Salmonella* Typhimurium DNA [26]).

For determination of Cq values, data from amplification curves were exported as .csv files and analyzed with the software environment R [27] with use of the qpcR package [28]. The function pcrbatch was used to fit the data for all curves with a five-parameter log-logistic function. Cq values were determined by the second derivate maximum method (called “cpD2” in the qpcR package). The amplification efficiency was calculated from individual amplification curves by application of the function described in [29]. For graphical visualization, GraphPad Prism version 6.0 was used. To determine the fluorescence intensity of ROX, ImageJ [30] was used, and intensity was measured within ten chambers per panel.

To determine when there was a significant effect on the number of positive reactions, a statistical tool developed by Sidstedt et al. [22] was used. The tool was applied to find any systematic differences in the probability of positive reactions between samples with or without inhibitor. The output is given as posterior mean of difference and posterior mass greater than zero, where 100% indicates that the means are different.

### Real-time PCR and gel electrophoresis

The qPCR experiments were all performed with a LightCycler Nano instrument (Roche Diagnostics) with a reaction volume of 20 μL. LightCycler Nano Software version 1.1 was used for determination of Cq values.

For the *invA* assay, the amplification conditions were 95 °C for 2 min, followed by 50 cycles of 10 s at 95 °C, 20 s at 60 °C, and 30 s at 72 °C. Unless otherwise stated, the following reagents were included in all master mixes for the *invA* assay: 1× Ex*Taq* buffer, 0.2 mM dNTP, 2.0 mM MgCl_2_ in total, each primer at 0.3 μM (InvA forward primer, InvA reverse primer [19]), 0.2 μM hydrolysis probe (InvA-minor groove binder [19]) or 1× EvaGreen (Biotium, Hayward, CA, USA), 1 U Ex*Taq* HS DNA polymerase, and 4 μL DNA (0.013 ng/μL).

For the *RB1* assay, the amplification conditions were 95 °C for 2 min, followed by 50 cycles of 10 s at 95 °C, 20 s at 60 °C, and 30 s at 72 °C. Unless otherwise stated, the following reagents were included in all master mixes for the *RB1* assay: 1× Ex*Taq* buffer, 0.2 mM dNTP, 4.0 mM MgCl_2_ in total, each primer at 0.3 μM (RB1_80F and RB1_235R [18]), 0.2 μM hydrolysis probe (RB1_212_MGB [18]) or 1× EvaGreen, 1 U Ex*Taq* HS DNA polymerase, and 2 μL DNA (1 ng/μL).

For the inhibitor experiments, the inhibitory compound was added to a master mix containing all the reagents described above, including DNA. If needed, Super-Q water (Merck, Darmstadt, Germany) was added to obtain a total volume of 20 μL per reaction.

PCR products were visualized by gel electrophoresis (1% agarose, stained with 1× GelRed from Biotium, 100 V, 30-min run time). Subsequently, images were aquired with BioOne Quantity (Bio-Rad, Hercules, CA, USA).

### Electrophoretic mobility shift assay

Binding reaction mixtures consisting of 1× PCR buffer [50 mM KCl, 10 mM tris(hydroxymethyl)aminomethane hydrochloride (Tris-HCl) pH 8, with 4 mM MgCl_2_ in total], 105 ng of genomic *Salmonella* Typhimurium DNA, and different amounts of IgG from 10 to 80 μg were prepared and incubated at room temperature for 1 h. Thereafter, 10 μL of the product was subjected to gel electrophoresis (1% agarose, stained with 1× GelRed, 100 V, 30-min run time). Subsequently, images were aquired with BioOne Quantity. For IgG and amplicons, the same workflow was applied, with 40 ng of *invA* assay amplicon DNA in the binding reactions. For hemoglobin, the same workflow was used, with different amounts of hemoglobin from 10 to 80 μg (genomic DNA only).

### Isothermal titration calorimetry

ITC is a powerful tool for both thermodynamic and kinetic studies in chemistry, and is applied, for example, in enzymatic studies. The principle of ITC is to measure the heat released or absorbed during a chemical reaction, and it has a significant advantage compared with other techniques in that it does not rely on any labeling or fluorescence detection as it is the heat from the enzymatic process itself that is measured. In this work we used a model system with Klenow fragment, which is active at 37 °C, since the ITC measurements are less stable at 72 °C.

The titration experiments were performed with a PEAQ-ITC instrument (MicroCal, Northampton, MA, USA) with a cell volume of 200 μL and a 40-μL syringe. Lyophilized deoxyadenosine triphosphate, deoxycytidine trisphosphate, deoxyguanosine triphosphate, and deoxythymidine triphosphate (Jena Bioscience, Germany) were dissolved in 1× PCR buffer (50 mM KCl, 10 mM Tris-HCl pH 8, and 4 mM MgCl_2_) to a stock concentration of 10 mM for each dNTP and then used at a concentration of 0.1 mM in the syringe. Protein concentration of Klenow fragment (product number EP0054, Thermo Scientific) was determined from the UV absorbance at 280 nm with a BioDrop μLITE instrument (BioDrop, Cambridge, UK) with a path length of 0.5 mm, a molar extinction coefficient of 55,450, and a molecular mass of 68 kDa. Oligonucleotides used as a primer–template pair were adapted from Datta and LiCata [31, 32]. The following oligonucleotides were purchased from Integrated DNA Technologies (Coralville, IA, USA) as high-performance liquid chromatography purified: 70mer, AAACCCTTGGACGGCTGCGAAAGTCGGCAAACGGCACGGTTATCCCAGTCACGAGCATGTACGCTGCGTA; 45mer, TACGCAGCGTACATGCTCGTGACTGGGATAACCGTGC CGTTTGCC.

Aliquots of 10 μL of dNTPs (0.1 mM) were injected into the cell (10 s per injection). The cell was stirred throughout the experiment (500 rpm) and the cell temperature was set to 37 °C. The cell contained 300 nM Klenow fragment and 1 μM 70mer and 45mer oligonucleotides as a template for the Klenow fragment in 1× PCR buffer (50 mM KCl, 10 mM Tris-HCl pH 8, and 4 mM MgCl_2_), with a total volume 500 μL. Control experiments where dNTPs were injected into the cell containing all of the reagents mentioned above except for the Klenow fragment were performed. For the inhibitor experiments, 0.50 μM hematin or 0.53 μM IgG was used in the reactions. Data analysis for enzyme kinetics was performed with PEAQ-ITC analysis software (MicroCal) for determination of Δ*H* (enthalpy), *K*_m_ (Michaelis–Menten constant) and *K*_cat_ (catalytic rate constant).

## Results

### Amplification in the presence of whole blood and specific blood components

The first objective was to evaluate inhibition effects of whole blood in a standardized dPCR setup using the DNA polymerase Ex*Taq* HS (Table 1). A general inhibitory effect was the lowering of the number of positive reactions, seen for 10% (v/v) whole blood (143 ± 130 positive reactions, compared with 417 ± 17 without blood). With 15% (v/v) blood, complete inhibition occurred. Evident with 10% (v/v) blood was also a decrease in amplification efficiency as measured from individual amplification curves (amplification efficiency 1.24 ± 0.13 compared with 1.91 ± 0.24 without inhibitor). There was no systematic increase in the Cq values. Notably, apart from the added purified DNA, some DNA is released from the white blood cells. Analysis of 2.5% (v/v) blood, without additional template DNA, resulted in 76 ± 13 positive reactions. However, with increased amounts of blood, the inhibitory effect surpassed the slight increase in DNA.

**Table 1:** Digital polymerase chain reaction results with different amounts of whole blood added to the standardized reactions. The *RB1* assay was applied with 50 ng human DNA added. A control reaction was set up with 2.5% blood (control 2.5) to determine how much amplifiable material is coming from the blood itself

Blood (%, v/v)	Positive reactions	DNA (ng/μL)	Cq value	Amplification efficiency
Control 2.5	76 ± 13	4.4 ± 0.8	23.03 ± 1.64	1.75 ± 0.19
0	417 ± 17	34.0 ± 2.1	25.10 ± 2.80	1.91 ± 0.24
2.5	438 ± 8	36.1 ± 1.0	24.57 ± 2.23	1.98 ± 0.23
5.0	478 ± 15	41.6 ± 2.2	22.56 ± 2.25	1.78 ± 0.12
7.5	489 ± 8	43.2 ± 1.1	23.92 ± 1.43	1.51 ± 0.17
10	143 ± 130	9.5 ± 9.3	28.04 ± 3.89	1.24 ± 0.13
15	5 ± 4	0.3 ± 0.2	NA	NA

The mean and standard deviation are shown for triplicate analyses

*Cq* quantification cycle, *NA* not applicable

**Fig. 1:**
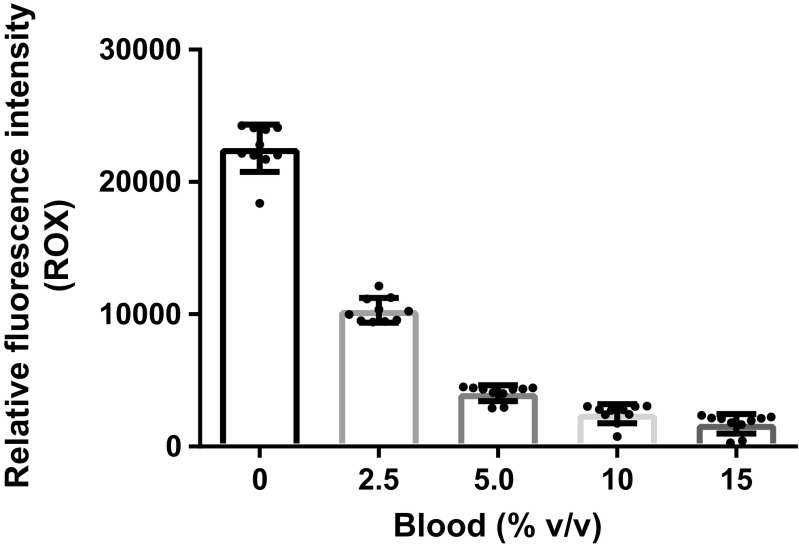
Quenching of the passive reference dye 6-carboxy-X-rhodamine (ROX) by whole blood. Relative fluorescence intensity means for 0–15% blood in the reactions are shown. The error bars represent standard deviation, and *n* = 10

Another effect of blood was quenching of the passive reference dye ROX, which was used to normalize the fluorescence from the probe applied to detect the target DNA (Fig. 1). The quenching effect was discovered since the amplification curves for reactions with blood had higher normalized fluorescence intensities than those without blood (see Fig. S1). With 2.5% (v/v) blood in the reactions, the ROX fluorescence was quenched to about 45% of the intensity without blood.

**Fig. 2:**
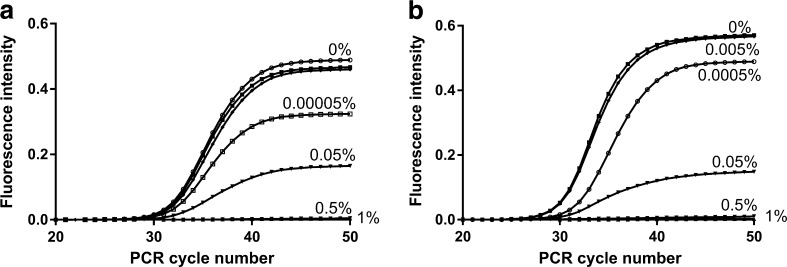
EvaGreen real-time polymerase chain reaction results with different amounts of whole blood in the reactions. Two different assays were applied, targeting either **a** the *invA* gene of *Salmonella enterica* serovar Typhimurium DNA with 0.052 ng DNA added or **b** the *RB1* gene of human DNA with 2 ng DNA added

In qPCR, with use of the same standardized setup as in dPCR but with application of the double-stranded DNA (dsDNA)-binding dye EvaGreen for detection, strong fluorescence quenching of EvaGreen was observed: 0.5% (v/v) blood gave flat amplification curves (Fig. 2), although gel electrophoresis showed that high amounts of amplicons were generated for up to 10% (v/v) blood (data not shown). These results collectively indicate that blood affects both amplification and fluorescence detection when applied in dPCR and qPCR.

In whole blood, many components can act as PCR inhibitors. To study the effect of potential inhibitors in blood separately, several proposed inhibitors were analyzed (i.e., EDTA, hematin, hemoglobin, heparin, IgG, FeCl_3_, and lactoferrin). Hemoglobin and IgG were found to be the most prominent inhibitors in both dPCR and qPCR (data not shown), as previously observed [8, 17].

**Fig. 3:**
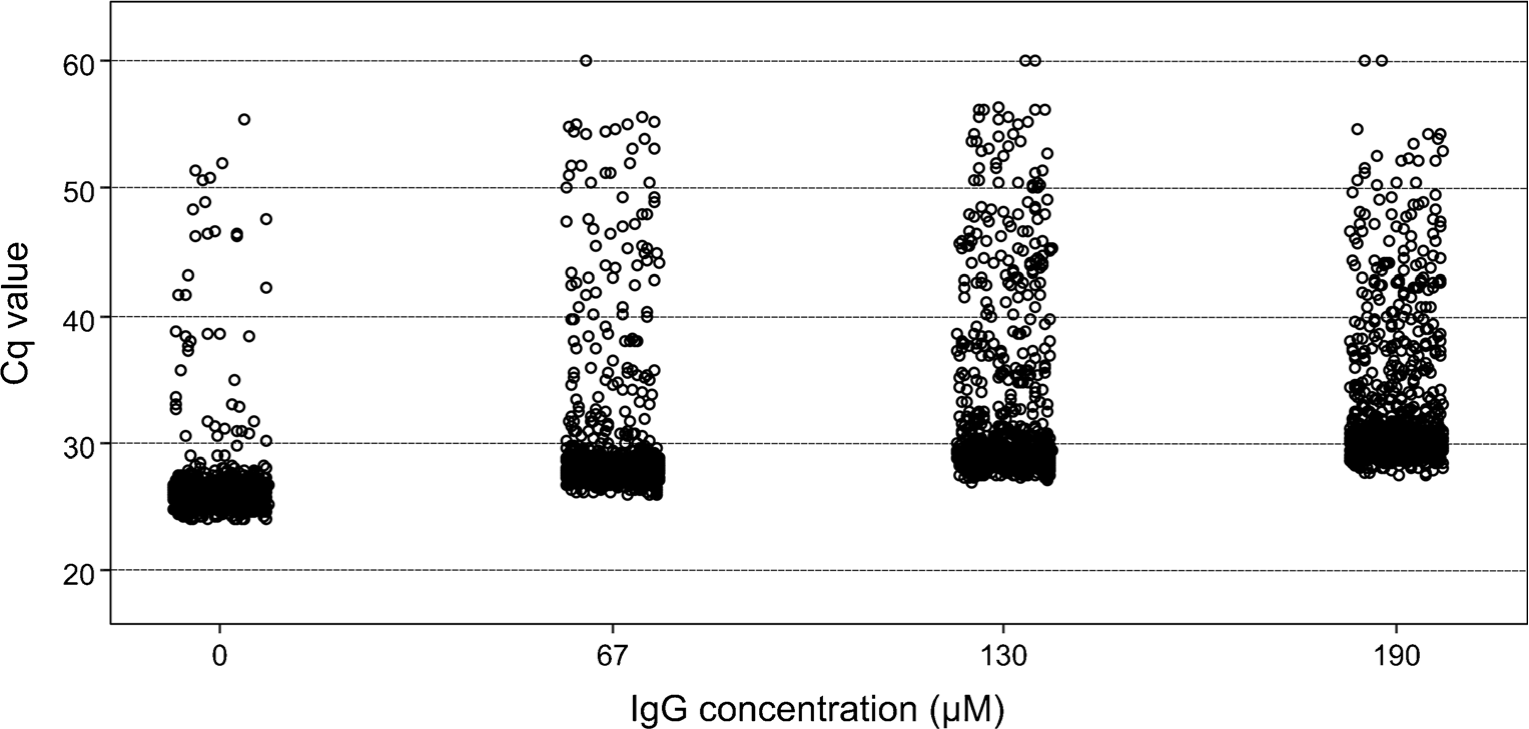
Resulting quantification cycle (Cq) values with immunoglobulin G (IgG) added to digital polymerase chain reaction reactions. An assay targeting the *invA* gene of *Salmonella enterica* serovar Typhimurium was applied, and 60 pg DNA was added to the reactions. For four amounts of IgG (0, 67, 130, and 190 μM), the Cq values for each positive reaction of three panels combined are shown, each circle representing one positive reaction

Inhibition effects caused by IgG were first studied in dPCR (Table 2). IgG mainly resulted in increased Cq values: 32.57 ± 5.60 with 190 μM IgG compared with 26.56 ± 3.19 without inhibitor. In addition, the Cq values were higher and showed greater variation with IgG added (Fig. 3), and the number of positive reactions was lowered: 352 ± 10 positive reactions with 27 μM IgG compared with 381 ± 11 without inhibitor (posterior mean of difference 0.04 and approximately 99.8% of posterior mass greater than zero, i.e., a result different from that with samples without inhibitor). Notably, with IgG in the reaction there was no apparent quenching of ROX and no impact on the amplification efficiency of individual amplification curves (1.29 ± 0.15 with 190 μM IgG compared with 1.42 ± 0.11 without inhibitor).

**Table 2:** Summary of results for immunoglobulin G (IgG) in digital polymerase chain reaction analysis. An assay targeting the *invA* gene of *Salmonella enterica* serovar Typhimurium was applied, and 60 pg DNA was added to the reactions

IgG (μM)	Positive reactions	DNA (pg/μL)	Cq value	Amplification efficiency
0	381 ± 11	45.47 ± 1.92	26.56 ± 3.19	1.42 ± 0.11
27^a^	352 ± 10	40.74 ± 1.55	27.47 ± 3.18	1.38 ± 0.11
40	320 ± 6	35.86 ± 0.82	28.68 ± 4.68	1.35 ± 0.13
53	310 ± 15	34.42 ± 2.23	29.54 ± 4.72	1.31 ± 0.11
67	336 ± 21	38.34 ± 3.18	28.88 ± 4.71	1.34 ± 0.12
110	311 ± 8	34.55 ± 1.15	31.61 ± 6.32	1.33 ± 0.14
130	310 ± 24	34.36 ± 3.48	32.15 ± 6.53	1.33 ± 0.12
160	310 ± 6	34.35 ± 0.90	33.16 ± 7.09	1.31 ± 0.12
190	308 ± 10	34.06 ± 1.38	32.57 ± 5.60	1.29 ± 0.15

The mean and standard deviation are shown for triplicate analyses

*Cq* quantification cycle

^a^The amount of IgG that led to a significant lowering of the number of positive reactions

**Fig. 4:**
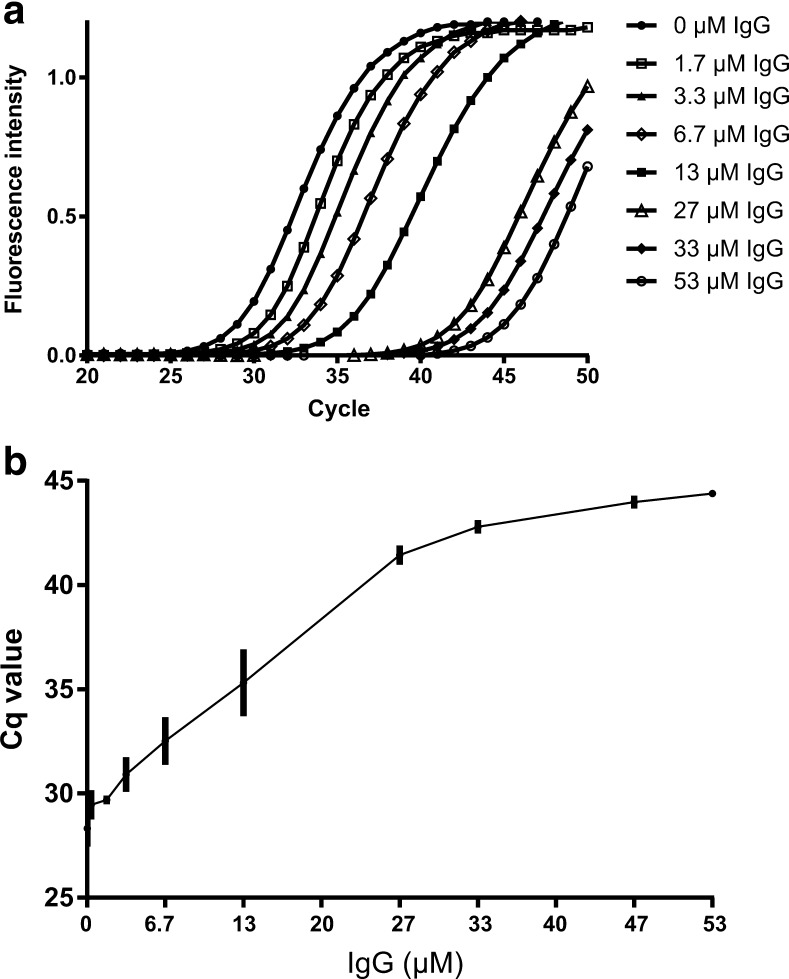
EvaGreen real-time polymerase chain reaction results with different amounts of immunoglobulin G (IgG). The assay targeting the *invA* gene of *Salmonella enterica* serovar Typhimurium was applied, and 52 pg DNA was added to the reactions. **a** Real-time polymerase chain reaction amplification curves with increasing amounts of IgG and **b** the generated Cq values, with error bars representing the standard deviation, *n* = 3. Cq quantification cycle

In qPCR, IgG caused a gradual increase in Cq values: for 53 μM IgG the Cq value was 44.4 ± 0.1 compared with 29.1 ± 0.2 without inhibitor (Fig. 4). As IgG caused delayed amplification but had no effect on measured amplification efficiency (slope), the amplification curves for increasing amounts of IgG resembled those from serial dilutions of DNA.

To investigate how hemoglobin affects dPCR reactions, hemoglobin, hematin, or FeCl_3_ was added to the reaction mixtures (see Fig. S2a for examples of amplification curves). Increasing amounts of hemoglobin caused an almost linear decrease in the number of positive reactions (Table 3). A significant decrease in the number of positive reactions occurred for 39 μM hemoglobin (posterior mean of difference of 0.04 and approximately 99% of posterior mass greater than zero). With 160 μM hemoglobin, the number of positive reactions was 273 ± 6 compared with 410 ± 12 without inhibitor (i.e., a reduction of more than 30%). As in the case with whole blood, quenching of the passive reference dye ROX was seen with hemoglobin. With 39 μM hemoglobin, the ROX fluorescence was quenched to about 50% of the intensity without hemoglobin (see Fig. S2b). There was also a decrease in amplification efficiency with increasing amounts of hemoglobin, similar to the effect of blood: 1.26 ± 0.10 with 470 μM hemoglobin compared with 1.68 ± 0.22 without inhibitor. No systematic increase in the Cq values was observed with hemoglobin (26.36 ± 5.20 with 470 μM hemoglobin compared with 25.37 ± 2.13 without inhibitor), but 620 μM hemoglobin caused complete amplification inhibition.

**Table 3:** Digital polymerase chain reaction results when hemoglobin, hematin, or FeCl_3_ was added to the reactions. The assay targeting the *RB1* gene of human DNA was applied with 50 ng DNA added

Hemoglobin (μM)	Positive reactions	DNA (ng/μL)	Cq value	Amplification efficiency	Molecules per chamber
0	410 ± 12	32.7 ± 1.5	25.37 ± 2.13	1.68 ± 0.22	0
39^a^	383 ± 2	29.5 ± 0.2	24.29 ± 1.07	2.10 ± 0.22	1.75 × 10^10^
78	347 ± 6	25.7 ± 0.6	24.24 ± 1.98	2.21 ± 0.21	3.50 × 10^10^
160	273 ± 6	18.8 ± 0.5	24.14 ± 2.30	1.99 ± 0.20	7.00 × 10^10^
310	139 ± 3	8.5 ± 0.2	24.32 ± 4.33	1.61 ± 0.15	1.40 × 10^11^
470	58 ± 8	3.3 ± 0.5	26.36 ± 5.20	1.26 ± 0.10	2.10 × 10^11^
620	0 ± 0	NA	NA	NA	–
Hematin (μM)	Positive reactions	DNA (ng/μL)	Cq value	Amplification efficiency	Molecules per chamber
0	410 ± 12	32.7 ± 1.5	25.37 ± 2.13	1.68 ± 0.22	0
1000	405 ± 7	32.0 ± 0.9	24.09 ± 3.06	1.79 ± 0.12	4.52 × 10^11^
2000	395 ± 19	30.9 ± 2.1	23.10 ± 2.54	1.44 ± 0.07	9.03 × 10^11^
3000	0 ± 0	NA	NA	NA	1.35 × 10^12^
4000	0 ± 0	NA	NA	NA	1.81 × 10^12^
6000	0 ± 0	NA	NA	NA	2.71 × 10^12^
FeCl_3_ (μM)	Positive reactions	DNA (ng/μL)	Cq value	Amplification efficiency	Molecules per chamber
0	410 ± 12	32.7 ± 1.5	25.37 ± 2.13	1.68 ± 0.22	0
1000	403 ± 24	31.9 ± 2.8	24.62 ± 2.08	1.89 ± 0.14	4.52 × 10^11^
2000	395 ± 5	30.8 ± 0.6	23.70 ± 1.56	2.06 ± 0.20	9.03 × 10^11^
3000	395 ± 24	30.9 ± 2.7	22.63 ± 1.97	1.82 ± 0.15	1.35 × 10^12^
4000^a^	357 ± 23	26.7 ± 2.3	26.28 ± 3.24	1.50 ± 0.11	1.81 × 10^12^
6000	0 ± 0	0.0 ± 0.0	NA	NA	2.71 × 10^12^

The mean and standard deviations are shown for three replicates

*Cq* quantification cycle, *NA* not applicable

^a^The amount of inhibitor that led to a significant lowering of the number of positive reactions

Hematin, a derivative of the heme group, is often used as a model for blood in forensic PCR inhibition experiments. In each hemoglobin molecule there are four heme groups. Thus, if heme is the sole agent causing PCR inhibition by hemoglobin, then four hematin molecules would be expected to have the same inhibitory effect as one hemoglobin molecule. However, 2000 μM hematin was less inhibitory than 470 μM hemoglobin (395 ± 19 positive reactions compared with 58 ± 8 positive reactions). Hematin caused a decrease in the number of positive reactions, and complete amplification inhibition was obtained with 3000 μM hematin (Table 3). Fluorescence quenching of the passive reference dye ROX was observed for hematin, similar to what was found for whole blood and hemoglobin. With 1000 μM hematin, the ROX fluorescence was quenched to about 25% of the intensity without hematin (see Fig. S2c). For hematin there was also a decrease in amplification efficiency: 1.44 ± 0.07 with 2000 μM hematin compared with 1.68 ± 0.22 without inhibitor. As for hemoglobin, there was no systematic increase in the Cq values (23.10 ± 2.54 with 2000 μM hematin compared with 25.37 ± 2.13 without inhibitor).

Each heme group contains one iron ion. From analysis of the inhibitory effect of FeCl_3_, we found 4000 μM FeCl_3_ resulted in a reduced number of positive reactions (357 ± 23 compared with 410 ± 12 without inhibitor, posterior mean of difference of 0.07 and approximately 100% of posterior mass greater than zero). With 6000 μM FeCl_3_, complete inhibition occurred. No fluorescence quenching of ROX was observed with FeCl_3_ (data not shown).

In qPCR, hemoglobin caused complete fluorescence quenching of EvaGreen at 1.6 μM although PCR product could be detected with gel electrophoresis up to 620 μM (see Fig. S3a, b). With hematin, 80 μM caused complete amplification inhibition, and fluorescence quenching of EvaGreen was observed with 50 μM (see Fig. S3c). With FeCl_3_, about 30 μM resulted in complete amplification inhibition, and no fluorescence quenching was observed (see Fig. S3d).

### PCR inhibition mechanisms of IgG and hemoglobin

**Fig. 5:**
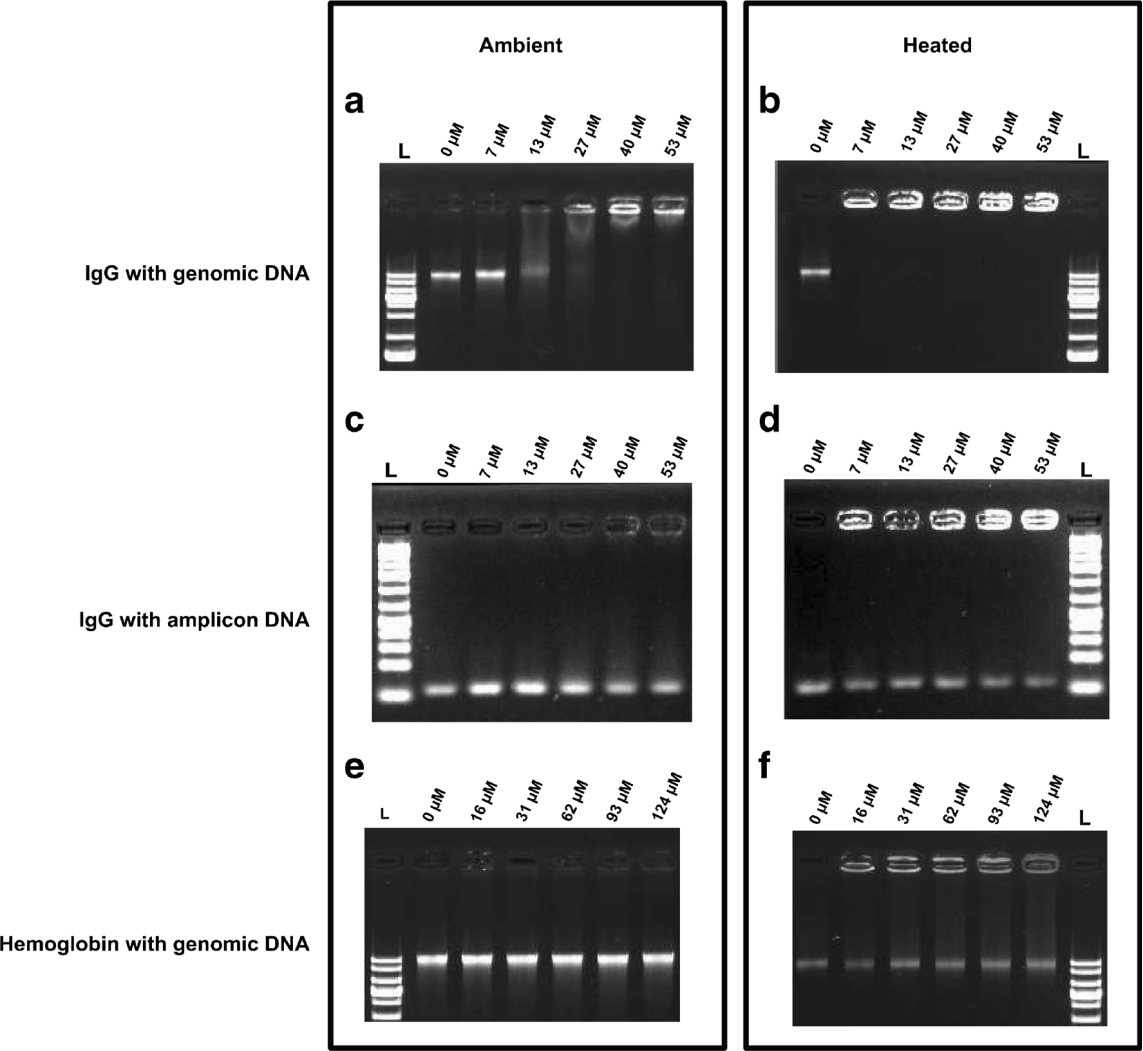
The binding between DNA and immunoglobulin G (IgG) or hemoglobin according to electrophoretic mobility shift assay analysis. Panel A 105 ng of genomic DNA was loaded per sample together with various amounts of IgG (no heating) and panel B the same experiment with heating at 95 °C for 30 s before analysis. Panel C 40 ng of amplicon DNA was loaded per sample together with various amounts of IgG (no heating) and panel D the same experiment with heating at 95 °C for 30 s before analysis. Panel E 105 ng of genomic DNA was loaded per sample together with various amounts of hemoglobin (no heating) and panel F the same experiment with heating at 95 °C for 30 s before analysis

IgG and hemoglobin were further studied to determine if they cause amplification inhibition through a direct effect on the DNA polymerase activity, by binding to DNA, or by a combination of the two. EMSA was used to investigate if IgG or hemoglobin binds to DNA (Fig. 5). The principle of EMSA is that if a protein binds to DNA, the mobility of the DNA in a gel will be altered. Without IgG, as well as with 7 μM IgG, the expected high molecular weight band was clearly visible on the gel (Fig. 5, panel A). However, for 13–27 μM IgG the band was fainter, indicating that IgG binds to the genomic DNA, thus hindering its migration in the gel. With 40–53 μM IgG, all the genomic DNA was retained in the well. When genomic DNA and IgG were heated together before EMSA analysis, to simulate the conditions in PCR, 7 μM IgG led to the DNA being retained in the well (Fig. 5, panel B). The same setup was repeated but with PCR amplicons (88 bp) and there, whether heated or not, the mobility of the amplicon DNA was only slightly affected (Fig. 5, panels C and D). This indicates that IgG has higher binding affinity for the large genomic DNA molecules than for the smaller amplicons. When we performed EMSA analysis with hemoglobin, no binding between the protein and genomic DNA was noted (Fig. 5, panels E and F).

**Fig. 6:**
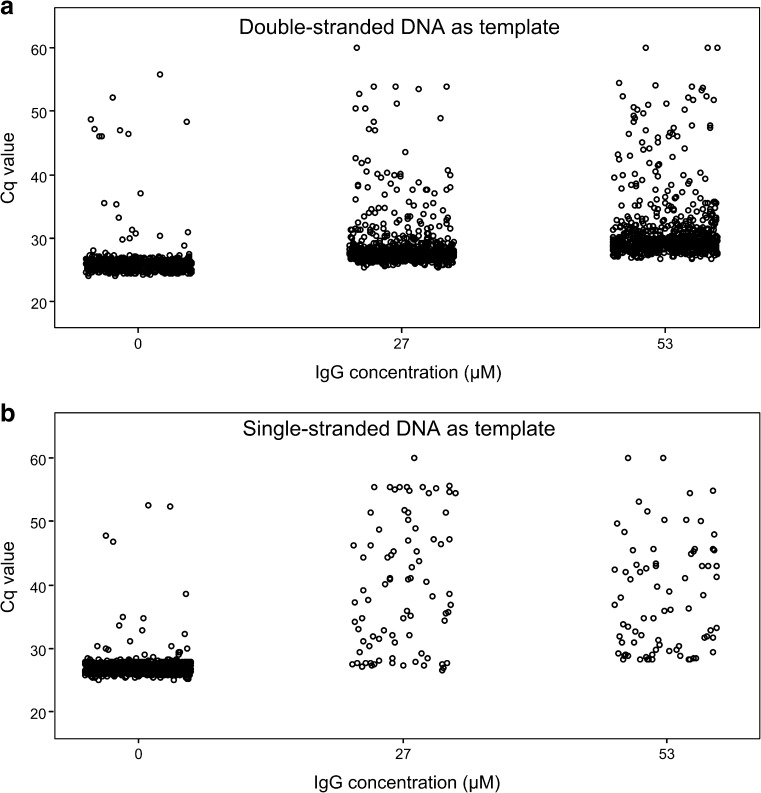
Immunoglobulin G (IgG)-induced inhibition in digital polymerase chain reaction with **a** double-stranded DNA or **b** single-stranded DNA as the starting template. An assay targeting the *invA* gene of *Salmonella enterica* serovar Typhimurium with 60 pg DNA added was applied. The quantification cycle (Cq) values for each positive reaction of three panels are shown for each IgG concentration, each circle representing one positive reaction

Next, the effect of IgG and hemoglobin when ssDNA or dsDNA was used as the starting template in dPCR was investigated to further investigate the nature of the inhibition. The results for IgG experiments are presented in Fig. 6 and Table S1. Amplification with ssDNA resulted in 26 ± 5 positive reactions with 27 μM IgG compared with 456 ± 9 without inhibitor. With dsDNA as the starting template, there were 269 ± 8 positive reactions with 27 μM dsDNA compared with 333 ± 8 positive reactions without inhibitor. For the *RB1* assay, 20 μM IgG resulted in 348 ± 13 positive reactions with dsDNA and 30 ± 5 with ssDNA (see Fig. S4 and Table S2). For hemoglobin, there was no difference between ssDNA and dsDNA (see Table S3). Amplification with 310 μM hemoglobin and dsDNA as the starting template gave 139 ± 3 positive reactions (posterior mean of difference of 0.35 compared with no hemoglobin), and with ssDNA 198 ± 8 positive reactions (posterior mean of difference of 0.40) (note that ssDNA generally gives a higher number of positive reactions as the number of template molecules is doubled compared with dsDNA).

With ssDNA as the starting template, amplification was almost completely inhibited by 10% whole blood (9 ± 9 positive reactions compared with 567 ± 8, posterior mean of difference of 0.72); that is, ssDNA template is more sensitive to inhibition by whole blood than is dsDNA (see Fig. S1 and Table S4).

**Fig. 7:**
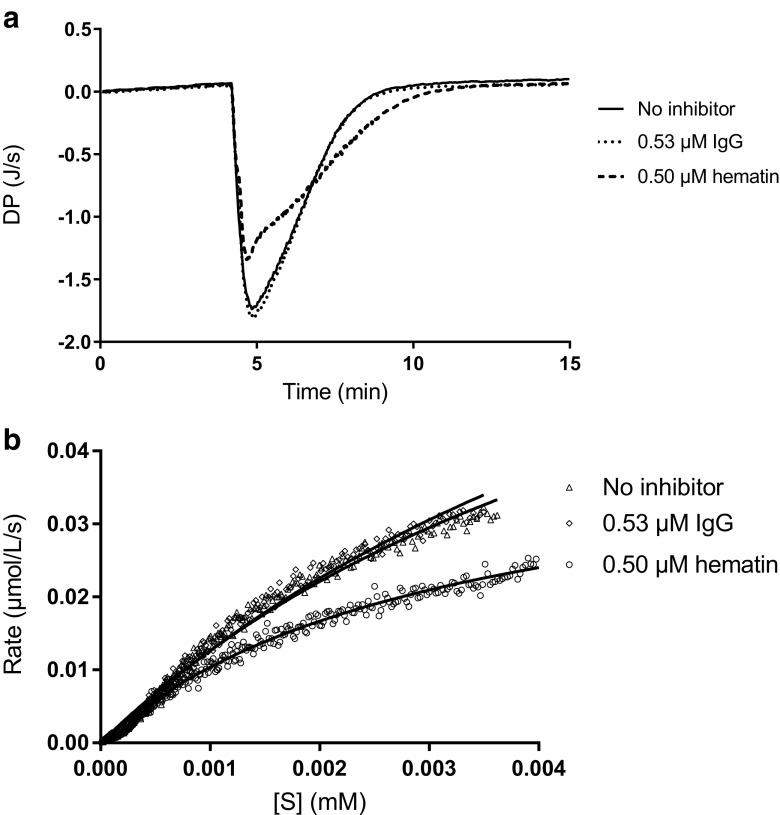
Isothermal titration calorimetry results where **a** the titration curves and **b** the data analyzed with PEAQ-ITC analysis software are shown. DP differential power, IgG immunoglobulin G

ITC was used to investigate how hematin and IgG affect the DNA polymerase activity by measurements of the DNA polymerization process in the absence/presence of hematin/IgG (Fig. 7, Table 4). A model system was applied, and we measured the polymerization by Klenow DNA polymerase at 37 °C with an oligonucleotide pair with a 70mer as the template and a 45mer as the primer. We found that 0.50 μM hematin had a negative impact on the DNA polymerase activity, observed as a lower *k*_cat_ (0.150 1/s compared with 0.345 1/s without inhibitor) and a slightly lowered *K*_m_ (3.13 μM compared with 6.07 μM without inhibitor). IgG seemingly did not affect *k*_cat_ or *K*_m_, and thus the activity of the Klenow DNA polymerase was not disturbed by IgG.

**Table 4:** Isothermal titration calorimetry results for the energetics and enzyme kinetics of Klenow fragment polymerization in the presence of inhibitors

	∆*H* (kJ/mol)	*k*_cat_ (1/s)	*K*_m_ (M)	Reduced *χ*^2^ [(μmol/L/s)^2^]
No inhibitor	-274.9	0.312	6.07 × 10^-6^	1.20 × 10^-6^
0.53 μM IgG	-276.6	0.367	7.31 × 10^-6^	1.10 × 10^-6^
0.50 μM hematin	-257.7	0.150	3.13 × 10^-6^	2.90 × 10^-7^

*IgG* immunoglobulin G

An increased amount of DNA polymerase (5 U instead of 1 U) was applied in dPCR to further study the effects of hemoglobin, hematin, and FeCl_3_ on the enzyme (see Table S5). More polymerase gave a slight improvement for hemoglobin, increasing the number of positive reactions for 470 μM hemoglobin to 160 ± 17 (posterior mean of difference of 0.31) with 5 U DNA polymerase compared with 58 ± 8 (posterior mean of difference of 0.46) with 1 U DNA polymerase. For 3000 μM hematin, amplification was recovered, going from zero positive reactions with 1 U DNA polymerase to 389 ± 8 (posterior mean of difference of 0.01 and approximately 82% of posterior mass greater than zero) with 5 U DNA polymerase. With 4000 μM FeCl_3_, the amplification was rather similar with 1 U or 5 U DNA polymerase: 347 ± 18 (posterior mean of difference of 0.07) compared with 357 ± 23 (posterior mean of difference of 0.07).

## Discussion

In this study, we have shown that IgG binds to genomic ssDNA, but has low affinity for binding to the much smaller amplicons. Likely, this is the cause of the delayed amplification seen as elevated Cq values in dPCR and qPCR when whole blood is analyzed. In 1979, the nature of anti-DNA activity of IgG from human serum of healthy people was studied, and it was found that a small portion of IgG binds to ssDNA [33]. In a study that focused on lupus monoclonal antibodies it was found that there are different binding mechanisms, where some antibodies bind to dsDNA alone and some preferably bind to dsDNA in a complex with a DNA polymerase [34]. The thermodynamics of antibody binding to DNA has been investigated with 20mer duplex DNA molecules. There it was observed that this fragment length allows only one arm of an IgG molecule to bind the DNA [35]. This means that IgG could potentially bind to smaller DNA fragments such as amplicons as well as to larger genomic DNA molecules. However, our results indicate that in the PCR, IgG interacts significantly more strongly with genomic DNA. In the initial PCR cycles, there is a screening phase where the primers localize and bind to the genomic target region. As the PCR continues, amplicons will be formed, serving as perfect templates for amplification. Amplification efficiency, measured when amplicons are the dominating target, is not reduced by the presence of IgG. Since it is mainly the Cq values that are affected, we hypothesize that IgG interferes with the reaction in the early PCR cycles, where genomic DNA is the dominating template. The inhibitory effect of IgG seemingly disappears when amplicons have become the dominating template, because of its lower affinity for binding to smaller DNA fragments.

Previously, it was found that PCR inhibition by different clones of IgG was increased when target DNA was heated together with IgG [17]. There it was speculated that inhibition was due to interaction with ssDNA. Our results prove this interaction, since IgG and whole blood had a much stronger negative effect when ssDNA rather than dsDNA was used as the starting template in the dPCR. Also, the EMSA experiments showed that heating of IgG and DNA, leading to ssDNA being formed, gave stronger interactions between IgG and DNA. When IgG denatures, it forms aggregates [36]. It is thus reasonable to assume that larger aggregates are formed in the larger reaction volume of qPCR (20 μL) compared with the smaller volume of dPCR (0.75 nL). The lower tolerance for IgG in qPCR could be explained by the inhibitory properties of large aggregates (53 μM in qPCR caused almost complete amplification inhibition, whereas 190 μM in dPCR did not completely hinder amplification).

Hemoglobin has been suggested to inhibit PCR by the release of iron ions [8]. Hematin is often used as a model for inhibition by blood [37–41]. In this study, these three inhibitors were applied separately, and differences among them were found. Release of iron ions is not likely the reason for inhibition by hemoglobin since we observed different effects from these two species when they were applied separately; for example, FeCl_3_ does not disturb the fluorescence detection or impact the amplification efficiency, and higher amounts are needed in dPCR for amplification inhibition (6000 μM FeCl_3_ versus 620 μM hemoglobin). The fact that FeCl_3_ does not affect the amplification efficiency implies that it does not disturb the DNA polymerase activity in the same way as hemoglobin and hematin. Our results also indicate that it is not only the heme that is responsible for hemoglobin-induced inhibition since we observed that there are differences between hematin and hemoglobin. Hemoglobin was shown to be a more potent inhibitor (i.e., lower amounts caused complete amplification inhibition in dPCR). Further, the fluorescence quenching was severer for hemoglobin than for hematin. In qPCR, lower amounts of hematin (80 μM) than hemoglobin (620 μM) caused amplification inhibition.

In qPCR with EvaGreen, hemoglobin mainly disturbs the detection by fluorescence quenching. Amplified product of the correct size could be observed up to 620 μM hemoglobin even though problems with detection occurred for 1.6 μM hemoglobin. This effect was also evident in dPCR since the dye ROX was severely quenched by hemoglobin. Previously, humic acid was also demonstrated to quench ROX fluorescence in dPCR [22]. In that study as well as in the present study, ROX quenching did not have an impact on the quantification. However, ROX quenching could cause problems for unknown samples since it could lead to inaccurate setting of the threshold distinguishing negative reactions from positive reactions, possibly leading to overestimation of the DNA concentration. Fluorescence quenching seemingly affects free dye molecules such as ROX and EvaGreen rather than the ones attached to probes (fluorescein in this case). This effect on free dye molecules was also observed for humic acid in qPCR, where static quenching was found to be a probable mechanism [16].

In structural biology, heme proteins were long excluded from protein fluorescence structural analysis because heme groups quench the emission of tyrosine and tryptophan residues [42]. A similar mechanism could be the reason for the fluorescence quenching by hemoglobin observed in this study, which is the first systematic investigation of fluorescence quenching by blood components in a PCR context. Previously, it was observed that blood causes quenching of the dye SYBR Green I [13, 43]. In a study where the toxicity of synthetic dyes was investigated, bovine hemoglobin was applied, and interactions were studied with spectroscopic techniques [44]. Among other dyes, fluorescein was studied, and the results indicated that the dyes bind within the central cavity of hemoglobin. This could explain why hemoglobin is a more potent fluorescence inhibitor than hematin. However, we did not observe any systematic quenching of fluorescein fluorescence in PCR, possibly because the fluorophore is attached to the probe, making it less accessible for the hemoglobin.

Previously, it was found that mutations leading to increased DNA binding affinity resulted in a higher tolerance to blood [13, 45]. Our ITC results indicate that hematin disturbs the DNA polymerase activity (Klenow), whereas IgG does not. Further studies of enzyme kinetics could be a good way forward in achieving a more detailed understanding of PCR inhibition mechanisms. In 1975 it was suggested that hemin interferes with the ability of DNA polymerase to bind DNA in erythroid cells by binding reversibly to the enzyme [46]. This was confirmed by another study performed more than 30 years later [13].

Direct PCR analysis of blood samples is very appealing since it would reduce the cost and the time to results. The work done to improve the blood tolerance in PCR to allow direct analysis has included finding alternative DNA polymerases, engineering DNA polymerases, and applying various facilitators [1, 2, 11–13, 43, 45, 47]. We have contributed to this field by elucidating the inhibitor mechanisms of blood and blood components in dPCR and qPCR. We have shown that whole blood causes several negative effects, such as quenching of fluorescence, reduced amplification efficiency, and a loss of amplifiable target DNA. By using several standardized inhibitors representing substances found in blood, we showed the inhibition in whole blood is mainly attributed to IgG and hemoglobin (Table 5). IgG has an effect on single-stranded genomic DNA template, disturbing amplification in the first few PCR cycles. Hemoglobin hinders amplification throughout the PCR process by a direct effect on the DNA polymerase activity, and also causes fluorescence quenching affecting amplicon detection and the passive reference dye ROX.

**Table 5.** Summary of the proposed inhibition mechanisms for the molecular inhibitors

Molecule	Inhibitor effect	Proposed mechanism/s
IgG	Increased Cq values and eventually complete amplification inhibition	Binds to genomic ssDNA, thereby hindering primer annealing or binding of DNA polymerase, thus disturbing the initiation of amplification in the first few cycles
Hemoglobin	Decreased amplification efficiency, leading eventually to complete amplification inhibition Quenching of fluorescence of free dye molecules (ROX, EvaGreen)	Lowers the activity of DNA polymerase throughout the PCR Binds to or interacts with fluorescent dyes, causing static fluorescence quenching
Hematin	Same as hemoglobin, but a weaker effect	Lowers the activity of DNA polymerase throughout the PCR Binds to or interacts with fluorescent dyes, causing static fluorescence quenching

*Cq* quantification cycle, *IgG* immunoglobulin G, *PCR* polymerase chain reaction, *ROX* 6-carboxy-X-rhodamine, *ssDNA* single-stranded DNA

## Electronic supplementary material

ESM 1 (PDF 903 kb)
