# Supplementary material for: Inhibition mechanisms of hemoglobin, immunoglobulin G, and whole blood in digital and real-time PCR
Source: Anal Bioanal Chem. 2018 Mar 5;410(10):2569–83. doi: 10.1007/s00216-018-0931-z (PMC5857286; doi:10.1007/s00216-018-0931-z)
Supplement: Supplementary file 1 — (PDF 903 kb) [file 216_2018_931_MOESM1_ESM.pdf]

Analytical and Bioanalytical Chemistry

Electronic Supplementary Material

Inhibition mechanisms of hemoglobin, immunoglobulin G and whole blood in digital and real-time PCR

Maja Sidstedt, Johannes Hedman, Erica L. Romsos, Leticia Waitara, Lars Wadsö,
Carolyn R. Steffen, Peter M. Vallone, Peter Rådström

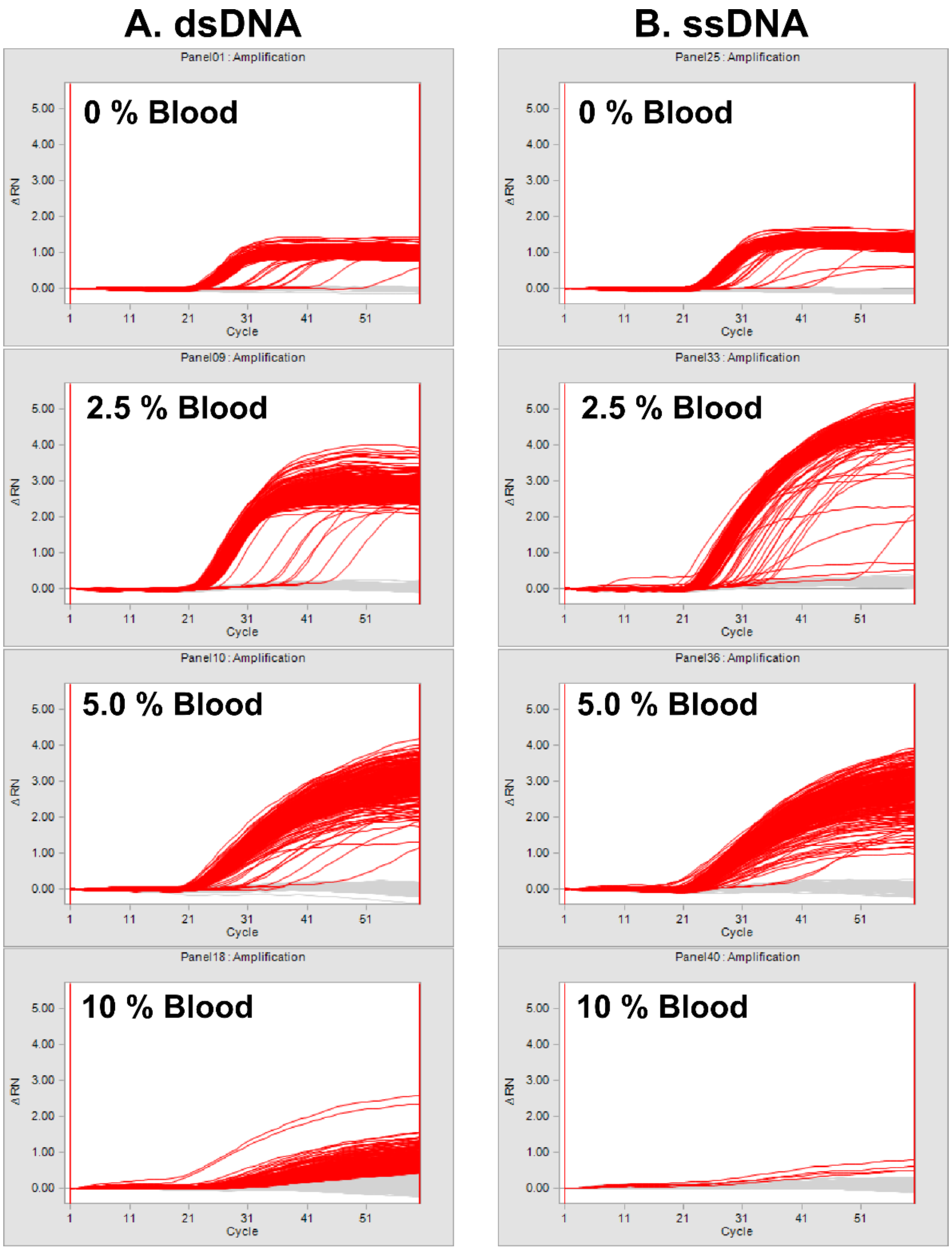

Fig. S1 Digital PCR results with different amounts of whole blood added to the reactions. An assay targeting the *rb1* gene of human DNA was applied with 50 ng DNA added. Examples of dPCR amplification curves with (**A**) dsDNA or (**B**) ssDNA as starting template

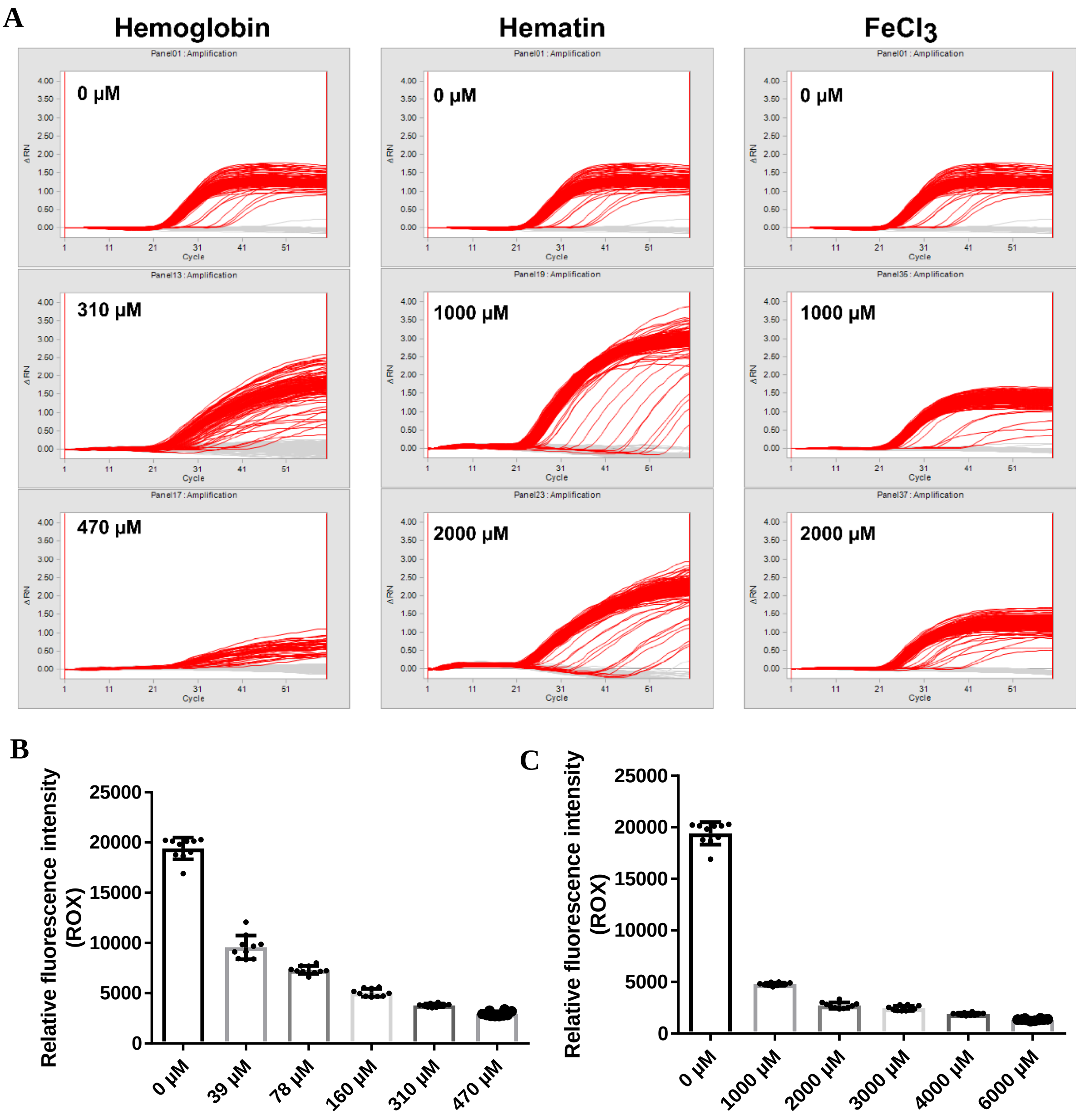

Fig. S2 (**A**) Example of amplification curves in prescence of hemoglobin, hematin or iron trichloride in dPCR using the assay targeting the *rb1* gene of human DNA with 50 ng DNA added. (**B**) ROX intensity with relative fluorescence intensity means (n=10) for 0 μM to 470 μM hemoglobin and (C) 0 μM to 6000 μM hematin

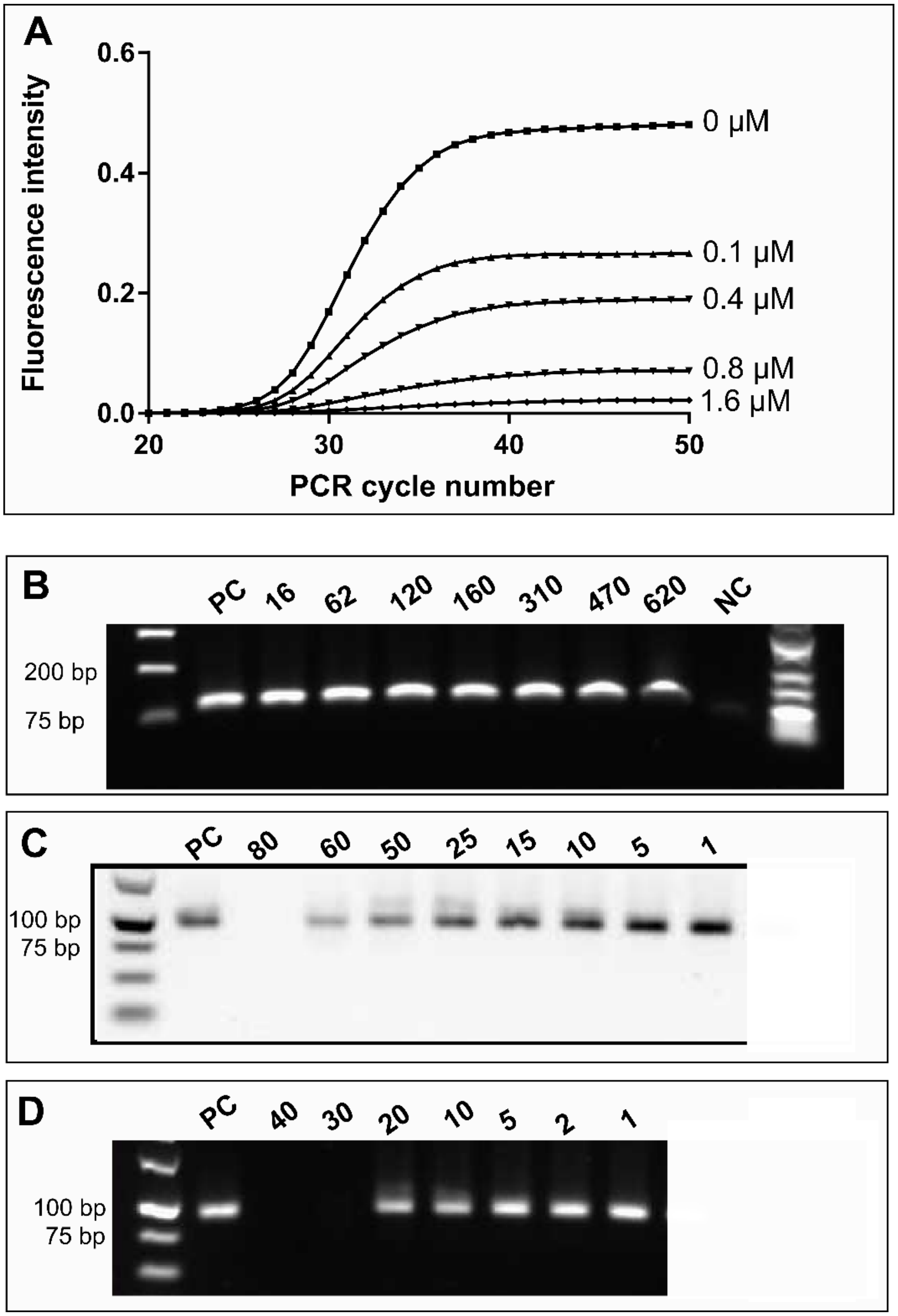

Fig. S3 EvaGreen qPCR results when the assay targeting the *invA* gene of *S.* Tm. And 0.052 ng DNA added with (**A**) 0 µM to 1.6 µM hemoglobin, and gel electrophoresis of PCR products for (**B**) 0 µM to 620 µM hemoglobin, (**C**) 0 µM to 80 µM hematin and (**D**) 0 µM to 40 µM iron trichloride. PC denotes sample without inhibitor and values the concentration (µM) of each inhibitor

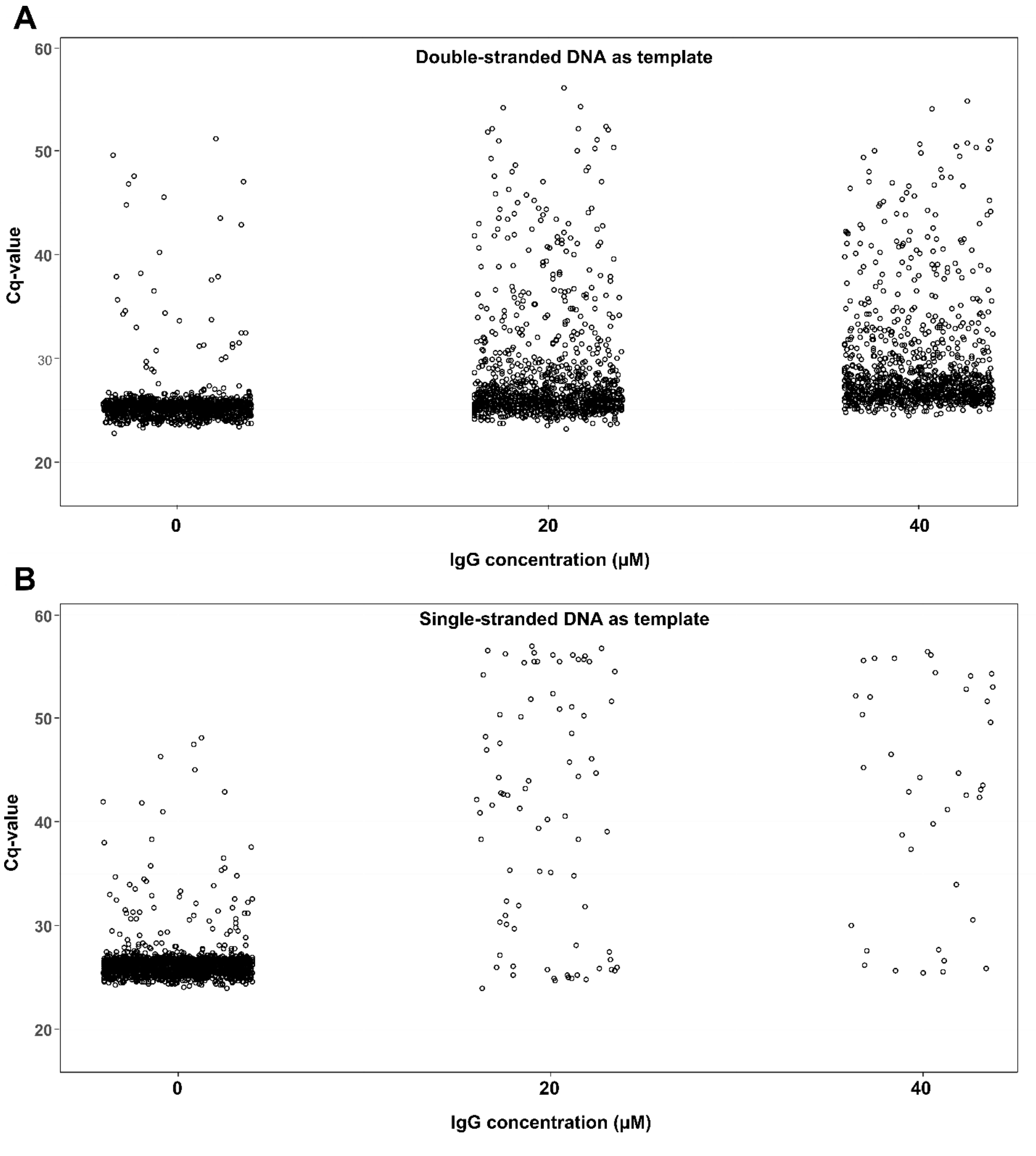

Fig. S4 IgG-induced inhibition in dPCR with (**A**) dsDNA or (**B**) ssDNA as starting template. An assay targeting the *rb1* gene of human DNA with 50 ng DNA added was applied. The Cq values for each positive reaction of three panels are shown for each IgG concentration, each circle representing one positive reaction

Table S1 IgG-induced inhibition in dPCR when using dsDNA versus ssDNA as template for the InvA assay in dPCR. An assay targeting the *invA* gene of S. Tm with 60 pg DNA added was applied with 0 µM, 27 µM or 53 µM of IgG. Results are shown as the means and standard deviations of three replicates

	**IgG amount (µM)**	**0**	**27**	**53**
**dsDNA**	Positive reactions	333 ± 8	269 ± 8	265 ± 10
	DNA concentration (ng/µL)	38.28 ± 1.15	28.70 ± 1.01	28.13 ± 1.25
	Cq value	26.15 ± 2.34	28.59 ± 3.83	30.94 ± 5.04
	Amplification efficiency	1.44 ± 0.05	1.33 ± 0.09	1.27 ± 0.10
**ssDNA**	Positive reactions	456 ± 9	26 ± 5	26 ± 5
	DNA concentration (ng/µL)	59.27 ± 2.64	2.29 ± 0.41	2.26 ± 0.41
	Cq value	27.20 ± 1.48	38.93 ± 9.63	36.45 ± 11.20
	Amplification efficiency	1.41 ± 0.06	1.38 ± 0.23	1.26 ± 0.18

Table S2 IgG-induced inhibition in dPCR with (**A**) dsDNA or (**B**) ssDNA as starting template. An assay targeting the *rb1* gene of human DNA with 50 ng DNA added was applied. Results are shown as the means and standard deviations of three replicates

	IgG amount (µM)	**0**	**20**	**40**
**IgG with dsDNA**	Positive reactions	405 ± 17	348 ± 13	356 ± 6
	DNA concentration (ng/µL)	32 ± 2	26 ± 1	27 ± 1
	Cq value	25.55 ± 2.33	28.29 ± 5.28	29.15 ± 4.92
	Amplification efficiency	1.75 ± 0.20	1.88 ± 0.34	1.80 ± 0.27
	IgG amount (µM)	**0**	**20**	**40**
**IgG with ssDNA**	Positive reactions	537 ± 9	30 ± 5	13 ± 4
	DNA concentration (ng/µL)	51 ± 2	2 ± 0	1 ± 0
	Cq value	26.36 ± 1.86	40.80 ± 11.46	42.63 ± 10.84
	Amplification efficiency	1.71 ± 0.14	1.94 ± 0.39	1.64 ± 0.24

Table S3 Amplification with ssDNA as template in presence of hemoglobin, hematin or iron trichloride. Digital PCR results with the assay targeting the *rb1* gene of human DNA was applied with 50 ng DNA added. Results are shown as the means and standard deviations of three replicates

**Hemoglobin**	Amount (µM)	**0**	**160**	**310**
	Positive reactions	506 ± 25	330 ± 7	198 ± 8
	DNA concentration (ng/µL)	46.0 ± 4.2	24.0 ± 0.7	12.8 ± 0.6
	Cq value	26.12 ± 1.70	24.77 ± 2.15	23.85 ± 2.59
	Amplification efficiency	1.78 ± 0.15	2.07 ± 0.26	1.77 ± 0.21
**Hematin**	Amount (µM)	**0**	**2000**	**3000**
	Positive reactions	506 ± 25	504 ± 8	113 ± 39
	DNA concentration (ng/µL)	46.0 ± 4.2	45.5 ± 1.2	6.9 ± 2.6
	Cq value	26.12 ± 1.70	24.02 ± 1.04	51.68 ± 7.41
	Amplification efficiency	1.78 ± 0.15	1.64 ± 0.09	1.06 ± 0.10
**$FeCl_3$**	Amount (µM)	**0**	**2000**	**4000**
	Positive reactions	506 ± 25	494 ± 8	217 ± 5
	DNA concentration (ng/µL)	46.0 ± 4.2	43.9 ± 1.3	14.2 ± 0.4
	Cq value	26.12 ± 1.70	25.02 ± 1.43	27.22 ± 5.65
	Amplification efficiency	1.78 ± 0.15	2.06 ± 0.22	1.29 ± 0.04

Table S4 Summary of dPCR results when starting with ssDNA as template and reactions in presence of whole blood. An assay targeting the *rb1* gene of human DNA was applied with 50 ng DNA added and results are shown as the means and standard deviations of three replicates. NA – not applicable

**Blood amount % (v/v)**	**Positive reactions**	**Cq value**	**Amplification efficiency**
0%	567 ± 8	26.23 ± 1.57	1.85 ± 0.16
2.5%	567 ± 14	23.70 ± 1.49	1.69 ± 0.20
5.0%	576 ± 12	23.28 ± 1.38	1.47 ± 0.25
7.5%	542 ± 84	26.26 ± 1.56	1.09 ± 0.07
10%	9 ± 9	NA	NA
15%	3 ± 3	NA	NA
only 5% blood	165 ± 8	23.38 ± 1.52	1.41 ± 0.16

Table S5 dPCR results with 5U DNA polymerase in presence of hemoglobin, hematin or iron trichloride. An assay targeting the *rb1* gene of human DNA was applied with 50 ng DNA added and results are shown as the means and standard deviations of three replicates

**Hemoglobin**	Amount (µM)	**0**	**310**	**470**
	Positive reactions	399 ± 10	250 ± 4	160 ± 17
	DNA concentration (ng/µL)	31.4 ± 1.2	16.8 ± 0.3	10.0 ± 1.2
	Cq value	24.61 ± 0.91	26.44 ± 2.12	26.60 ± 2.86
	Amplification efficiency	2.16 ± 0.18	1.56 ± 0.18	1.36 ± 0.13
**Hematin**	Amount (µM)	**0**	**2000**	**3000**
	Positive reactions	399 ± 10	404 ± 10	389 ± 8
	DNA concentration (ng/µL)	31.4 ± 1.2	31.9 ± 1.1	30.2 ± 0.9
	Cq value	24.61 ± 0.91	26.43 ± 1.82	24.74 ± 1.25
	Amplification efficiency	2.16 ± 0.18	1.45 ± 0.11	1.36 ± 0.11
**$FeCl_3$**	Amount (µM)	**0**	**3000**	**4000**
	Positive reactions	399 ± 10	413 ± 53	347 ± 18
	DNA concentration (ng/µL)	31.4 ± 1.2	33.3 ± 6.7	25.7 ± 1.8
	Cq value	24.61 ± 0.91	25.96 ± 0.80	29.53 ± 3.22
	Amplification efficiency	2.16 ± 0.18	1.56 ± 0.09	1.55 ± 0.19
